# Genes involved in mitochondrial biogenesis and function may not show synchronised responses to mitochondria in shell gland of laying chickens under infectious bronchitis virus challenge

**DOI:** 10.1186/s12860-019-0190-7

**Published:** 2019-04-01

**Authors:** Samiullah Khan, Juliet Roberts, Shu-Biao Wu

**Affiliations:** 1 Animal Science, School of Environmental and Rural Science, University of New England, Armidale, New South Wales 2351 Australia; 2 Present address: School of Animal and Veterinary Sciences, The University of Adelaide, Roseworthy, South Australia 5371 Australia

**Keywords:** Laying chicken, Mitochondrial function, Infectious bronchitis, Nuclear DNA encoded genes, Chicken oviduct

## Abstract

**Background:**

Egg formation takes place in the oviduct of laying hens over a 24 h period. Infectious bronchitis virus (IBV) causes pathological lesions in the chicken oviduct. In the current study, mitochondrial counts were determined in three different segments of the oviduct during egg formation in laying chickens challenged with IBV T strain. Nuclear DNA encoded genes that are involved in mitochondrial biogenesis, fission and function were studied in the shell gland of the oviduct undergoing virus multiplication.

**Results:**

In the shell gland, the mitochondrial count was significantly lower (*P* < 0.05) in the challenged group, compared with the control group. However, it did not vary in response to IBV challenge in the isthmus and magnum regions of the oviduct. The gene succinate dehydrogenase complex, subunit A, flavoprotein variant (*SDHA*) was down-regulated in the shell gland by IBV challenge (*P* < 0.05), while other genes being studied did not show responses to the challenge (*P* > 0.05). Differential expression of the genes was observed at different time-points of egg-shell formation. The expression levels of citrate synthase (*CS*), cytochrome C, somatic (*CYC, S*) and sodium-potassium adenosine triphosphatase (*Na*^*+*^*-K*^*+*^*ATPase*) genes were significantly higher, while those of *SDHA* and dynamin related protein 1 (*Drp1*) genes were significantly lower, at 15 h compared with 5 h following oviposition of the previous egg. The expression level of peroxisome proliferator-activated receptor gamma coactivator 1-alpha (*PGC-1α*) did not show significant change at different time-points.

**Conclusions:**

It was concluded that IBV T strain infection in laying hens reduced mitochondrial counts only in the shell gland region of the oviduct. The genes involved in mitochondrial biogenesis or function may not show synchronised responses to that of mitochondria in the shell gland of chickens under T strain of IBV challenge.

## Background

The infundibulum, magnum, isthmus and shell gland (uterus) contribute to egg formation in laying chickens. Egg-shell formation takes place in the isthmus and shell gland regions, where the former contributes only to shell membrane formation. Egg formation involves the production of steroid hormones, which regulate the proliferation and growth of oviducal epithelial cells throughout the oviduct. For example, the administration of estrogen and/or progesterone leads to massive growth of the epithelia in the oviduct of juvenile hens [1–3]. An egg is composed of egg internal contents and egg-shell. Among the internal contents, albumen is secreted by the magnum and is composed mainly of ovalbumin, muco-proteins and globulins [4]. The shell membranes are synthesized in the isthmus region of the oviduct and contain collagen proteins in its composition [5]. Egg-shell synthesis occurs in the shell gland and is composed mainly of calcium carbonate [6] and shell matrix proteins, such as the ovocalyxin family [5].

Mitochondrion acts as a powerhouse of the cell, where it plays a vital role in cellular metabolism, calcium handling, heme biosynthesis, cell differentiation, apoptosis and aging [7–9]. Mitochondrial count in a cell varies in different cell types [10, 11], different organs, the sex and age of an organism [12, 13] and physiological and disease conditions [10, 14]. A higher mitochondrial respiratory capacity and mitochondrial counts have been recorded in more physically active humans [15, 16]. Similarly, increases in mitochondrial DNA (mtDNA) have been observed during myogenesis in rats [17] and in brown adipocytes of various mammals in response to low temperature [18]. During the cell cycle, mitochondria undergo changes in shape, count and location; however, it is not known how quickly mitochondria divide in metabolically active and inactive cells. In the reproductive track of laying chickens, it is unclear how mitochondria respond to the need of the cells for ATP by either inducing mitochondrial division or accelerating mitochondrial function without division. Cells can respond to alterations in mitochondrial function by up- or down-regulations in the expression of nuclear-DNA encoded gene [19]. Mitochondrial transcription factor A (*mtTFA*) encodes a protein that in conjunction with nuclear-DNA polymerase γ (POLγA) control mtDNA replication in a cell [20, 21]. mtDNA replication machinery synthesizes different proteins that include the single-stranded DNA binding protein (mtSSB), the catalytic subunit of DNA POLγA and processivity factor, the mitochondrial RNA polymerase and the mitochondrial replicative helicase TWINKLE [22, 23]. Dynamin related protein 1 (Drp1 also called DNM1L) is the main regulator of mitochondrial division in a cell, which is oligomerized by mitochondrial division protein 1 (Mdv1) bound to the outer membrane of mitochondria via Mitochondrial fission 1 protein (*FIS1*) [24–27]. Nuclear genome of the cell encodes proteins essential for mtDNA maintenance and replication [23]. The synthesised proteins are translocated into the mitochondria after their packaging in the cytoplasm. Therefore, expression of genes coded by mtDNA and nuclear genomes are accurately coordinated for regulating the electron transport linked phosphorylation capacity in response to changes in physiological demands of cells [28]. Studies in a single celled green algae (chlorella) [29], HeLa cells (strain- F315) [30] and the opportunistic pathogenic dimorphic yeast (*Candida albicans*) [31] indicate that increase in total mitochondrial count per cell occurs continuously during the cell cycle. This increase in mitochondrial counts is usually positively correlated with the increase in cell volume. In HeLa and dimorphic yeast cells, mitochondrial count is comprised of approximately 10% of the total cell volume while, in algae, this value is about 3% but is constant throughout cell division [29–31].

Citrate synthase (*CS*) gene encodes an enzyme that is localised in the mitochondrial matrix after being shaped in the ribosome [32]. In mammals, the *CS* gene has been used extensively as a marker for intact mitochondrial density [33–35]; however, its role has been questioned in studies of developmental stages [36], age of animal [37] and physical activity [38]. *CS* has been shown to be positively correlated with acute exercise activities in mammalian muscles [39, 40]. Succinate dehydrogenase complex, subunit A (*SDHA*) gene, encodes a major catalytic subunit of succinate-ubiquinone oxidoreductase, a complex of the mitochondrial respiratory chain. *SDHA* gene provides instructions for synthesizing one of four parts (subunits) of the succinate dehydrogenase (SDH) enzyme. SDH then participates in both the electron transport chain and the Krebs cycle. Peroxisome proliferator-activated receptor gamma coactivator 1-alpha **(***PGC-1α/PPARGC1A*) is the master regulator in mitochondrial division [41]. In vitro studies in muscle cells [42], C2C12 myoblasts [43] or in mice cardiac myocytes [44] have shown that PGC-1α is capable of activating the expression of a cascade of genes involved in mitochondrial synthesis and respiratory function in different types of cells. Therefore, *PGC-1α* is mainly involved in mitochondrial energy metabolism and mitochondrial biogenesis. Cytochrome C somatic (*CYC, S*) gene encodes cytochrome C enzyme that plays a role in the electron transport chain complex in mitochondria and during apoptosis. *Na*^*+*^*-K*^*+*^ *ATPase* gene encodes an enzyme that is an integral membrane protein which consists of α and β subunits [45]. This enzyme plays an essential role in maintaining the transmembrane gradient of Na^+^ and K^+^ ions in cells [46].

Infectious bronchitis virus (IBV) is a highly contagious avian mucosal pathogen that not only affects layer and broiler chickens but also other avian species worldwide [47]. Similar to other coronaviruses, IBV is composed of a small membrane protein (E), the integral membrane protein (M), the nucleoprotein (N) and the spike glycoprotein (S) [48, 49]. The S protein is composed of two subunits, the S1 (head) and the S2 (stalk) that is responsible for binding to the target cell receptor in host [50, 51], while the N protein induces cell mediated immunity [52]. There is not much information about whether IBV entry and fusion to host cells occurs following endocytosis or at the cell membrane [51, 53]. Host cell neutral pH is considered lethal for fusion of the virus particle [54]. Among other host cell surface receptors, sialic acid has been shown to act as a receptor for binding of IBV spike proteins in respiratory, kidney and oviduct epithelia [55–58]. IBV can infect any age of birds; however, the mortality is higher in very young chicks compared with older birds [59]. Mortality rates depend upon viral strain, birds age and immune status of the bird [60]. Among all the field strains of Australian IBV, T (N1/62) is considered the most virulent in inducing pathological changes in the tracheal, kidney and oviduct epithelia of laying hens. Infection with IBV in the oviduct leads to various degrees of pathogenesis in the oviduct and reduction in egg production [61–65].

The present study aimed to: a) determine mitochondrial counts in the cells of oviduct segments in laying hens at different time-points of egg formation in relation to the requirement of energy for egg production during IBV challenge; b) to determine the expression of nuclear DNA encoded genes in the shell gland to gain insights into their responses to IBV infection and time-points of egg-shell formation.

## Methods

### Birds rearing

Day-old Isa-Brown laying chickens were vaccinated with Rispens vaccine for Marek’s disease but were not vaccinated for infectious bronchitis. At the University of New England, the chickens were raised in IBV free isolation sheds following strict biosecurity protocol. The birds were reared as per the guidelines of the ISA General Management Guide 2009–10. From the isolation sheds, pullets (18-week old) were moved to cages in an isolated poultry house. During the rearing period, no morbidity or mortality was recorded until the birds were challenged with IBV. Before IBV challenge, an ELISA (ELISA kit, IDEXX Laboratories, Inc., Westbrook, MA, USA) was performed on blood serum of all the chickens. At 35-week of flock age, eggs were processed for egg quality parameters following the method of Samiullah et al. [66]. Chickens were allocated into treatment groups (Table 1) by 2 × 2 factorial design based on egg-shell colour (L*) and egg weight (g) that were not significantly different (*P* > 0.05) among the treatment groups (data not shown).

**Table 1:** Allocation of birds into various groups for IBV challenge study in the oviduct of laying hens

Group	Time-point (hr)	
	5	15
IBV T strain (20 birds)	10 birds	10 birds
Control (20 birds)	10 birds	10 birds

Time-point (hr) refers to egg post-oviposition time as determined by video camera. At the time of processing of individual hen, the forming egg was either in the distal magnum/isthmus (5 h time-point) or in the shell gland (15 h time-point)

The hens selected for the virus inoculation were transferred to a separate layer cage house one week before the challenge in order for the chickens to adapt to the new shed and recover from the translocation stress. In both the virus challenge and the control groups, five chickens from each time-point at one time were inoculated intra-occularly with either 10^7^ embryo infective dose (E.I.D_50_)/bird of allantoic fluid or mock infected (PBS). The challenged chickens were closely observed for the development of clinical signs of IB [65] and loss of egg-shell colour until days 9–10 post-infection (p.i.). In a separate experiment, the E.I.D_50_ dose was calculated from virus being titrated in embryonated SPF-eggs. The incubated eggs were inoculated at day-9 with 10^− 3^ to 10^− 8^ serial dilutions of IBV T strain (N1/62) [67]. On day-16 of incubation, the eggs were opened and the number of dead, live or IBV affected embryos recorded after the method of Reed and Muench [68]. The virus infection in the chickens was confirmed through quantitative PCR (in oviduct tissue) and by ELISA of blood plasma samples.

### Oviduct tissue collection

For accuracy of sampling, the oviposition times for individual hens were recorded by video camera. At specific post-oviposition times (5 or 15 h), hens were euthanized with CO_2_ gas and approximately 500 mg pieces of tissue from each of the shell gland, isthmus and distal magnum were aseptically collected into RNALater. The tissue samples were stored in a − 20 °C freezer until processed for DNA/RNA extraction.

### DNA extraction

For the total genomic (including mitochondrial) DNA extraction from the oviduct tissue (all tissue layers), the TRIsure (Bioline, Australia) protocol was slightly modified for obtaining pure nucleic acids. Briefly, a 100 mg of tissue was thoroughly homogenized in 1 mL of TRIsure. For obtaining quality DNA/RNA, the TRIsure added samples were maintained on ice and the homogenisation step was also performed in a tube containing ice. For total RNA extraction, after the chloroform step, the transparent upper layer was used (see below). For DNA extraction, the remaining middle and bottom layers were thoroughly mixed and further processed. To precipitate DNA, 0.3 mL absolute ethyl alcohol was added into each sample and the samples were incubated for 3 min at room temperature. To obtain a DNA pellet, the ethanol precipitated DNA samples were centrifuged for 5 min at 2000×*g* in an Eppendorf centrifuge (4 °C). The resultant DNA pellet was washed with continuous shaking in 1 mL of 0.8 M sodium citrate (containing 10% absolute ethanol). The samples were centrifuged as described earlier. Next, the DNA pellet was washed in 1.5 mL of 75% ethyl alcohol and the samples centrifuged as described earlier. After maximum ethanol removal, the DNA pellet was dissolved in 100 μL TE buffer and the samples centrifuged for 10 min at 12000×*g* to remove any insoluble material. For concentration and purity measurements, the total DNA was analysed in a Nanodrop-8000 (ThermoFisher Scientific, USA). The average 260/280 and 260/230 ratios for individual DNA samples were in the acceptable range (1.8~2.2). The DNA samples were stored at − 20 °C until used for quantitative PCR assays.

### Total RNA extraction and purification

During DNA extraction, the upper layer after chloroform treatment was processed for total RNA extraction according to the manufacturer’s instructions (TRIsure protocol). The RNA was precipitated with 0.5 mL chilled iso-propanol and the pellet washed with 1.5 mL of 75% ethanol. The RNA pellet was dissolved in 100 μL of PCR grade water (RNase/DNase free) and processed for RNA purification using an RNeasy Mini Kit (Qiagen, Australia) as per the manufacturer’s instructions. The purified total RNA was analysed in a nanodrop as described earlier. RNA quality and concentration were further analysed in an Agilent 2100 Bioanalyzer using the Agilent RNA-6000 Nano Kit as per the manufacturer’s protocol. All the samples showed distinct 18S and 28S bands with an average RIN of ≥9.1. To quantify the viral RNA from the oviduct segments, epithelial scrapings were also processed for total RNA extraction following a partial modification of the method described by Chousalkar et al. [69]. Approximately 1 g of oviduct scrapings (magnum, isthmus and shell gland segments) was mixed with 1 mL of sterile PBS, shaken vigorously and centrifuged at 4800×*g* for 10 min at 4 °C. From the supernatant, 200 μL was mixed with 1 mL of TRIsure and total RNA was extracted as per the manufacturer’s protocol. The extracted total RNA was used for viral RNA quantification from oviduct tissue scrapings.

### Primer design and validation

The primer sequences shown in Table 2 were designed using NCBI-primer software with the design of at least one primer sequence spanning the exon-intron junction or the amplicon of the primers spanning over two exons with the intron in-between at size of at least 500 bp. For sequence specificity, the primers were cross checked in Ensemble Chicken Galgal4, NCBI database using BLASTN and UCSC’s Chicken (*Gallus gallus*) Genome Browser Gateway. Prior to real-time qPCR analysis, the primer specificity and amplification efficiency were determined in serial dilutions (10^− 1^ to 10^− 8^) of the purified RNA/DNA. The primer amplification efficiency (%) was analysed based on the mathematical equation [70]: E = 10^(1/slope)^ – 1.

**Table 2:** Forward (F) and reverse (R) primer sequence details used in the current study

Gene name	Abbreviation used	Primer sequence (5′ – 3′)	Amplicon size (bp)	Annealing temperature °C	Amplification efficiency (%)	R^2^	Slope	Accession No.
Glyceraldehyde-3-phosphate dehydrogenase	*GAPDH* ^*a*^	F-GGTCACCAAGAAGGTGGAGA R-GACAGTGCCCTTGAAGTGTC	137	63	96	0.99888	−3.434	NC_006088.3
NADH dehydrogenase subunit 4	*ND4* ^*b*^	F-CGCAGGCTCCATACTACTCG R-TTAGGGCACCTCATAGGGCT	137	60	99	0.99944	−3.341	NC_001323.1
Citrate synthase	*CS*	F-TACTACACGGTGCTCTTCGG R-CGGATCCTGCCGGATTTGTAG	152	60	96	0.99943	−3.089	XM_015300287.1
Succinate dehydrogenase complex flavoprotein subunit A	*SDHA*	F-TCTGTCCATGGTGCTAATCG R-TGGTTTAATGGAGGGGACTG	126	60	94	0.99790	−3.484	NM_001277398.1
Dynamin-1-like protein	*Drp1/DNM1L*	F-TGTGACCCGAAGACCCCTTA R-AGCATCTATCTCATTTTCATCTCCA	91	60	99	0.99861	−3.345	NM_001079722.1
Cytochrome C, somatic	*CYC, S*	F-CCCAGTGCCATACGGTTGAA R-CTCACCCCAAGTGATACCTTTGT	140	60	96	0.9930	−3.430	NM_001079478.1
PPARG coactivator 1 alpha	*PGC-1α/PPARGC1A*	F-AGTGACATCGAGTGTGCTGCT R-GGTCAAGTTCTGGGAGATCTGGG	70	63	100	0.99472	−3.331	NM_001006457.1
(Na^+^-K^+^) ATPase	*(Na* ^*+*^ *-K* ^*+*^ *) ATPase* ^*^	F-GTCAACCCGAGGGATGCTAA R-ACTGCTACAATGGCACCCTG	179	60	99	0.99663	−3.338	J03230.1
S1 glycoprotein	IBV T	F-TCAGGTGGTTGGCATTTACA R-ATTGCGAACTTGACCATTCC	181	60	99	0.99982	−3.323	U29522.1
Nucleocapsid protein (3′ UTR)	IBV Vic S	F-ATAGGCATGTAGCTTGATTACC R-GTTTCCAGGCTACTAAGTAGAC	76	60	98	0.99823	−3.320	DQ059623.1
TATA-Box Binding Protein	TBP*	F-TAGCCCGATGATGCCGTAT R-GTTCCCTGTGTCGCTTGC	147	61	97	0.99676	−3.407	NM_205103
Tyrosine 3-monooxygenase/tryptophan 5-monooxygenase activation protein, zeta polypeptide	YWHAZ*	F- TTGCTGCTGGAGATGACAAG R- CTTCTTGATACGCCTGTTG	60	60	104	0.99912	−3.222	NM_001031343.1

^a^Gene was used to amplify fragment of genomic DNA; ^b^Gene was used to amplify fragment of mtDNA

*The primer sequences for IBV Vic S [69], *TBP* [86], *YWHAZ* [87] and *(Na*^*+*^*-K*^*+*^*) ATPase* [88] were sourced from literature

### DNA cloning for mitochondrial count quantification

For mtDNA copy number quantification through qPCR, the 137 bp fragments of each of the *GAPDH* and *ND4* genes were ligated into plasmid vector using the Rapid One Shot chemical transformation protocol of the TOPO TA Cloning Kit for sequencing (ThermoFisher Scientific, Australia) as per the manufacturer’s protocol. Next, the recombinant plasmid was transformed into One Shot chemically competent *Escherichia coli* cells*.* A 50 μL of the cells was plated on Difco Luria-Bertani (LB) Agar (Bacto Laboratories, Australia) and incubated overnight at 37 °C. From the grown colonies, a single colony was enriched in LB broth and incubated overnight at 37 °C. From the cultured broth, 1 mL was used to extract recombinant plasmid DNA using the PureLink Quick Plasmid Miniprep Kit (ThermoFisher Scientific, Australia) as per the manufacturer’s protocol. The eluted recombinant plasmid DNA purity and concentration were analysed in the nanodrop as described earlier. The recombinant plasmid DNA samples were stored at − 20 °C until used for qPCR assays. To check the amplification efficiencies for the standard curve construction of *GAPDH* and *ND4*, qPCR was run on the recombinant plasmid DNA (10^− 2^~10^− 8^) in a 20 μL PCR reaction. The master-mix preparation and qPCR cycling conditions were as per the protocol of the SensiFAST SYBR No-ROX Kit (Bioline, Australia). For standard curve construction, eight different serial dilutions (10^− 2^~10^− 9^) of the recombinant plasmid DNA were prepared and qPCR amplified. To construct the standard curves for *GAPDH* and *ND4*, quantification cycles (Cq) were plotted against log_10_ copy number of plasmids calculated according to the molecular weight of recombinant plasmids [71]. The amplified PCR products were sequenced by the Australian Genome Research Facility, Australia for confirmation of *GAPDH* and *ND4* fragment inserts into the plasmid vector.

### Mitochondria quantification

For mitochondrial count per cell enumeration, mtDNA copies in a cell were normalised with genomic DNA copies [71, 72]. The SYBR green method of the SensiFAST SYBR No-ROX Kit (Bioline, Australia) was followed for qPCR as per the manufacturer’s protocol. The qPCR was performed in a Rotor-Gene Q thermocycler (Qiagen, Australia) in a total volume of 20 μL master-mix reaction. The reaction consisted of 10 μL 2× SensiFAST SYBR No-ROX mix, 6.4 μL RNase-free PCR grade water, 0.8 μL each of the primers and 2 μL of the DNA (10^− 2^ dilution of the extracted DNA). To rule out external contamination, a negative control reaction with no DNA template was included in each qPCR run. For standard curve construction and mitochondrial count per cell calculation, recombinant plasmid DNA dilutions (10^− 2^~10^− 9^) were included in the respective qPCR runs. The conditions for a 2-step qPCR were: denaturation at 95 °C for 3 min, 40 cycles of denaturation at 95 °C for 5 s and annealing and extension at 60 °C or 63 °C for 30 s (Table 2). Fluorescent data were acquired at the end of each annealing/extension step during qPCR cycles (40). The mtDNA copy number per cell in the magnum, isthmus and shell gland was calculated according to the equation: (mtDNA copies)/(gDNA copies/2) [71].

### Relative gene expression analysis

For the relative expression of gene studies, shell gland RNA samples were run in duplicate with the inclusion of appropriate internal controls. The qPCR master-mix was prepared as per the manufacturer’s protocol of SensiFAST SYBR Lo-ROX One-Step RT-PCR Kit (Bioline, Australia). The PCR final 20 μL volume reaction contained 10 μL of 2× SensiFAST SYBR low-Rox one-step mix, 0.4 μL of RiboSafe RNase inhibitor, 0.2 μL of reverse transcriptase, 0.8 μL of each of the forward and reverse primers, 3.8 μL RNase-free PCR grade water and 4 μL of RNA template (10^− 2^ dilution). Using a QIAgility robotic, the reaction volume was distributed into Rotor-Gene Disc 100 (Qiagen, Australia) and run in a Rotor-Gene Q thermal cycler. The two-step PCR conditions were: reverse transcription at 45 °C for 10 min, polymerase activation and denaturation at 95 °C for 2 min, 40 cycles of denaturation at 95 °C for 5 s and annealing and extension at 60 °C, 61 °C or 63 °C (according to Table 2) for 20 s. The fluorescent data collection, melting curve analysis and amplification efficiency calculation were performed as described previously.

### Viral RNA quantification from oviduct tissue

IBV T strain (kindly provided by CSIRO, Geelong, Australia) was cultured in 11-day-old SPF embryonated eggs and allantoic fluid was harvested at day-16 of incubation. Viral RNA was extracted from the allantoic fluid using TRIsure as per the manufacturer’s protocol. A 181 bp fragment of viral RNA was amplified using the SensiFAST SYBR Lo-ROX One-Step RT-PCR Kit as per the manufacturer’s instructions and cloned (using Rapid One Shot chemical transformation protocol of TOPO TA Cloning Kit for sequencing) into a plasmid vector for standard curve construction. Details of the cloning method have been described in a previous section. A standard curve was constructed from 10-times serial dilutions (10^− 2^~10^− 9^) of recombinant plasmid DNA cloned with 181 bp fragment of IBV T strain. Approximately 1 μg RNA from individual samples extracted from the magnum, isthmus and shell gland whole tissue (all tissue layers) or mucosal scrapings was reversely transcribed into cDNA using the QuantiTect Reverse Transcription Kit (Qiagen, Australia). Quantitative PCR was performed with the SYBR green method using the SensiFAST SYBR No-ROX Kit. Quantitative PCR reaction was performed in a total volume of 20 μL in duplicate on a Rotor-Gene Q thermal cycler and cloned plasmid DNA was included in the same run as standards. The cloned plasmid DNA with insert was calculated as number of copies per μL in six different dilutions for standard curve construction. Plasmid copy number was calculated based on the concentration of plasmid DNA and its molecular weight [71]. Individual sample amplicons from all the three segments of the oviduct were run in the Bioanalyzer as described earlier, to assess the specificity and size of the virus nucleic acid fragment. Viral load was calculated as cDNA copies per μL of PCR reaction volume multiplied by the total reaction volume, RNA elution volume and weight of the tissue used for RNA extraction. Viral load was expressed as viral cDNA copy number per gram of oviduct tissue.

### Statistical analysis

To determine the mitochondrial count per cell, the mtDNA copies per cell were analysed in Statview software (SAS Institute Inc., Version 5.0.1.0) taking the time-point and virus challenge as main effects. The statistical significance (*P* < 0.05) between mean values was determined by the Tukey-Kramer test. For gene expression data analysis, raw Cq values were analysed in qbase+ software version 3.0 against *TBP* and *YWHAZ* as reference genes [73]. The analysis was based on relative expression (2^-ΔΔCq^) using gene specific amplification efficiencies [74–76]. To determine the effect of time-point and IBV challenge, from the qbase+, normalised relative quantities (NRQ) were exported and analysed in Statview Version 5.0.1.0 (SAS Institute Inc., 1998) using one- and two- way ANOVA. Level of significance (*P* < 0.05) between the mean values was determined by Tukey-Kramer test.

## Results

**Fig. 1:**
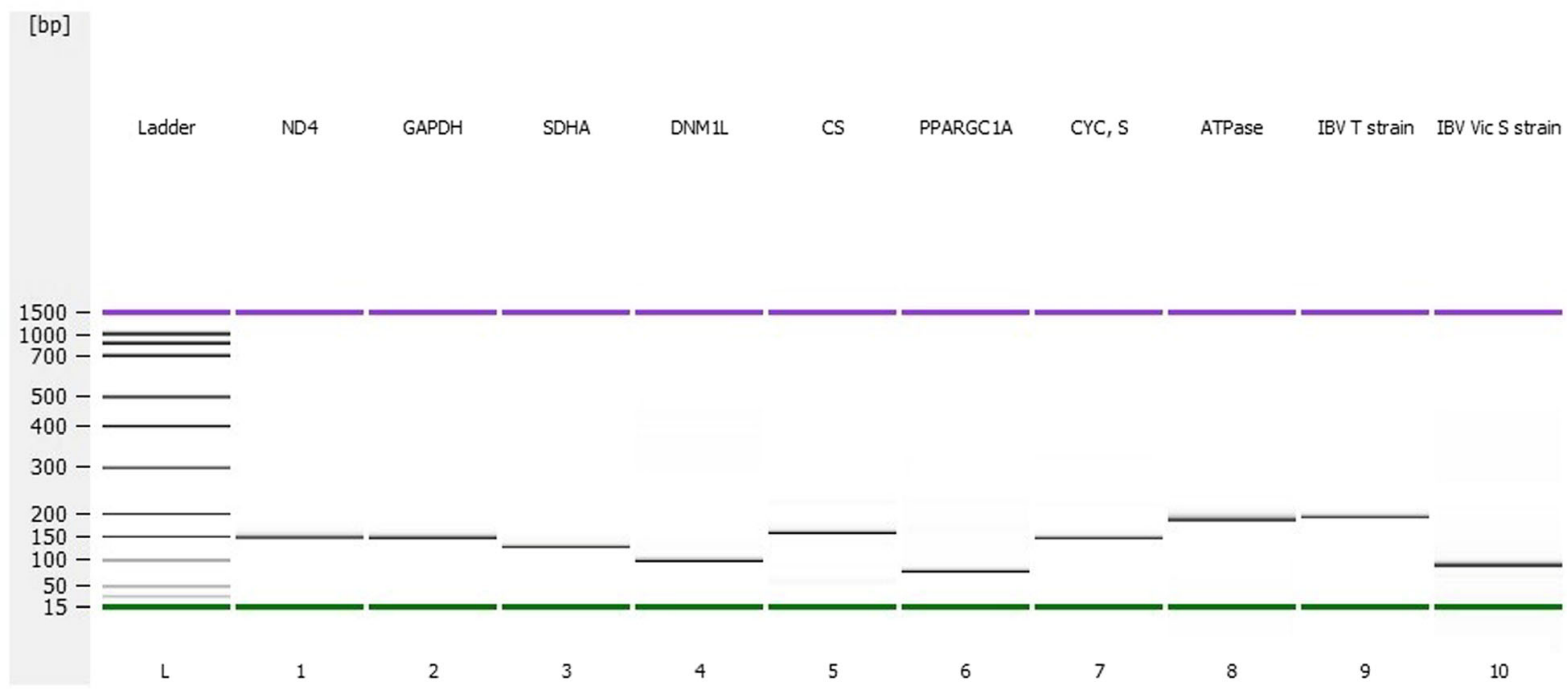
Gel electrophoresis for assessing the specificities of the primers. L) DNA ladder; 1) *ND4* (137 bp); 2) *GAPDH* (137 bp); 3) *SDHA* (126 bp); 4) *Drp1/DNM1L* (91 bp); 5) *CS* (152 bp); 6) *PGC-1α/PPARGC1A* (70 bp); 7) *CYC, S* (140 bp); 8) *Na*^*+*^*-K*^*+*^ *ATPase* (179 bp); 9) IBV T strain (181 bp); 10) IBV Vic S strain as a positive control (76 bp). For the DNA gel electrophoresis in the Agilent 2100 Bioanalyzer, Agilent DNA 1000 Kit was used as per the manufacturer’s instructions

All the primers used in this study were specific in amplifications of the expected products (Fig. 1). The amplification efficiency of the primers ranged from 94 to 104% (Table 2).

### Viral RNA quantification from oviduct

**Fig. 2:**
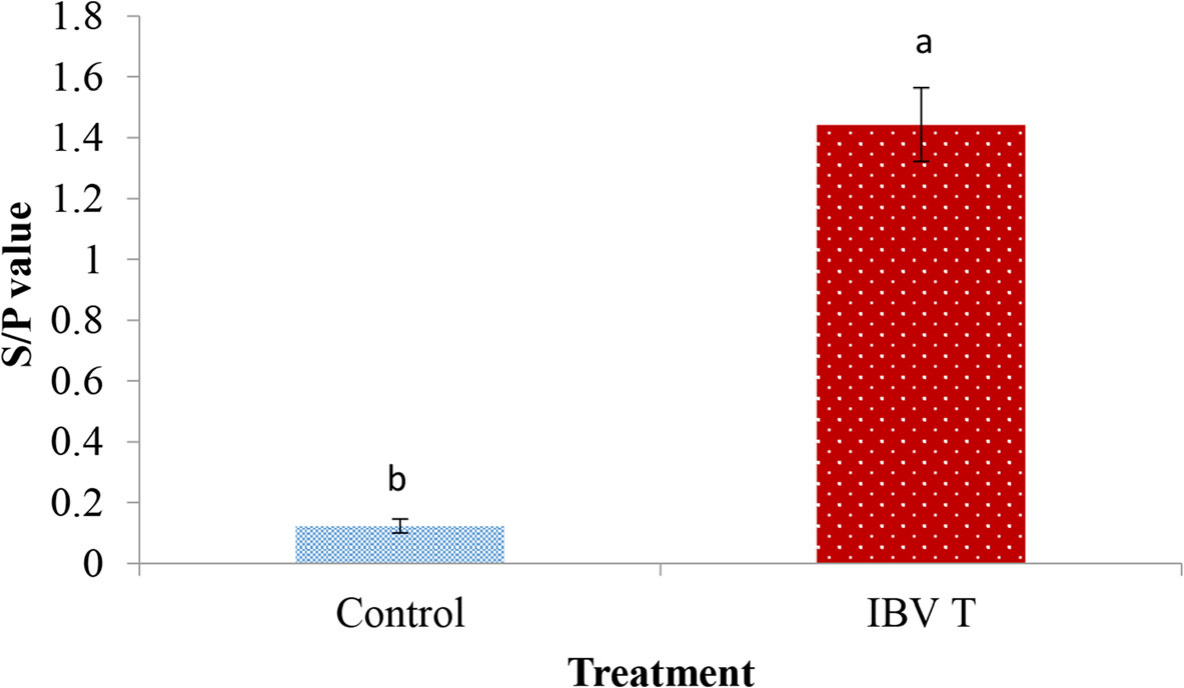
Antibody titre of the control and challenged hens on days 9–10 post-infection. The antibody titre was calculated as per the protocol of the Infectious Bronchitis Virus Antibody Test Kit (IDEXX Laboratories). According to the kit protocol, Sample/Positive (S/P) ratio > 0.20 shows antibody level positive for IBV. Bars represent standard deviation. Superscripts (^a,b^) show significant differences

IBV T RNA was not detectable in any of the three segments of the oviduct (all tissue layers) samples processed for real-time qPCR. However, viral RNA was detected in the shell gland epithelial scrapings. All samples from challenged birds were positive with a mean viral load of approximately 3.55 × 10^6^ copies per gram of shell gland tissue. Viral RNA was not detected in the epithelial scrapings of the magnum and isthmus segments of the oviduct of any of the challenged birds. A significantly higher titre of antibodies for the challenged birds in the ELISA test confirmed that the virus multiplied and caused a significant immune response in the challenged birds (Fig. 2). During the post-challenge period, all infected birds showed characteristic clinical signs of IB. No viral RNA was detected in the control birds as expected.

### Mitochondrial quantification

**Fig. 3:**
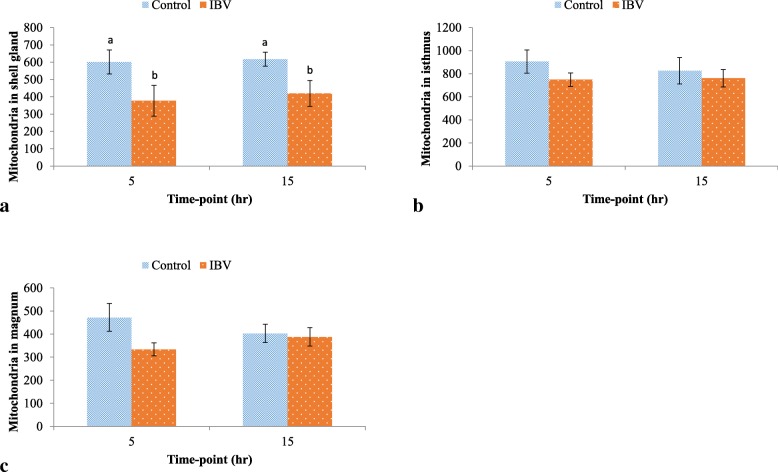
Mean mitochondrial count per cell in different segments of oviduct of laying hens at different time-points and IBV challenge. **a** Mitochondria in the shell gland (*P* = 0.0050). **b** Mitochondria in the isthmus (*P* = 0.2577). **c**. Mitochondria in the magnum (*P* = 0.0879). Superscripts (^a,b^) show significant difference between the control and IBV T strain challenged groups. Bars represent standard error of the mean values

The mitochondrial count in the shell gland region of the oviduct was significantly lower (*P* < 0.05) in the IBV T strain challenged group compared with the control group (Fig. 3a). IBV T challenge did not significantly affect mitochondrial counts in the isthmus and magnum regions of the oviduct (Fig. 3b, c). The mean mitochondrial count (per cell) was not significantly affected (*P* > 0.05) by time-points of egg formation in any of the three segments of the oviduct (Fig. 3). There was no significant interaction between the time-point and IBV T strain challenge for mitochondrial counts in the shell gland, isthmus and magnum regions of the oviduct.

### Effect of IBV challenge and time-point on gene expression in the shell gland tissue

**Fig. 4:**
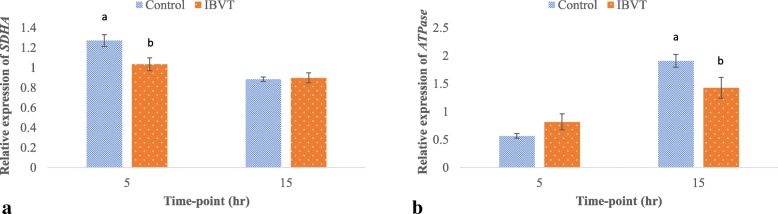
Relative expression levels of *SDHA* and *Na*^*+*^*-K*^*+*^ *ATPase* in the shell gland tissue affected by virus challenge and egg-shell formation time-point interaction. **a**. *SDHA*. **b**. *Na*^*+*^*-K*^*+*^ *ATPase.* Values are normalised relative quantities (NRQ) to the mean expression level of all the samples ± S.E. Superscripts (^a,b^) across the bars indicate significant difference in the viral challenged and control groups

Significant interactions (*P* < 0.05) between IBV challenge and time-point of egg-shell formation were observed for the expression of *SDHA* and *Na*^*+*^*-K*^*+*^ *ATPase* (Table 3; Fig. 4). The relative expression level of *SDHA* was significantly lower in the virus challenged groups compared with the control group at the 5 h time-points, whereas this effect was not observed at the 15 h time-point of egg-shell formation. The relative expression level of *Na*^*+*^*-K*^*+*^ *ATPase* was significantly lower in the virus challenged compared with the control group at the 15 h time-point, whereas at the 5 h time-point, this effect was not significant (Fig. 4a, b). The relative expression levels of all of the genes except the *SDHA* were not significantly affected (*P* > 0.05) by IBV challenge (Table 3). The expression levels of all the genes except *PGC-1α* were significantly affected by time-point of egg-shell formation. The expression levels of *CS*, *CYC, S* and *Na*^*+*^*-K*^*+*^ *ATPase* genes were significantly higher, while those of *SDHA* and *Drp1* genes were significantly lower, at the 15 h compared with the 5 h time-point of egg-shell formation (Table 3).

**Table 3:** Relative expression stabilities of genes in the shell gland of laying hens challenged with IBV

Gene	Group				*P* value		
	Virus challenge		Time-point (hr)		Virus challenge	Time-point	Interaction
	Yes	No	5	15			
*CS*	1.125 ± 0.097	1.050 ± 0.096	0.892 ± 0.092^y^	1.283 ± 0.080^x^	0.5522	0.0032	0.7135
*SDHA*	0.966 ± 0.042^b^	1.079 ± 0.054^a^	1.154 ± 0.026^x^	0.891 ± 0.050^y^	0.0343	< 0.0001	0.0188
*Drp1*	1.066 ± 0.063	0.993 ± 0.053	1.191 ± 0.044^x^	0.868 ± 0.047^y^	0.2619	< 0.0001	0.7039
*PGC-1α*	1.025 ± 0.053	1.019 ± 0.049	1.089 ± 0.062	0.956 ± 0.030	0.9273	0.0671	0.9236
*CYC, S*	0.959 ± 0.025	1.041 ± 0.021	0.829 ± 0.037^y^	1.247 ± 0.049^x^	0.1900	< 0.0001	0.5145
*Na* ^*+*^ *-K* ^*+*^ *ATPase*	1.123 ± 0.134	1.237 ± 0.165	0.692 ± 0.077^y^	1.668 ± 0.120^x^	0.3916	< 0.0001	0.0087

Values are normalised relative quantities (NRQ) to the mean expression level of all the samples ± S.E. Superscripts (^a,b^) across the row indicate significant difference between the virus challenged and control groups. Superscripts (^x,y^) across the row indicate significant difference between the 5 and 15 h time-points of egg-shell formation. In each group at each time-point, there were 10 samples processed for qPCR assay

## Discussion

We investigated the mitochondrial counts in three different segments of the oviduct of laying hens challenged with IBV T strain, at two stages of egg formation. A significantly lower mitochondrial count in the shell gland of challenged hens suggests that the virus had a greater effect on this region, as compared with the isthmus and magnum segments of the oviduct. A greater severity of pathological lesions caused by IBV T in the isthmus and shell gland segments, compared with the magnum, has been reported [77]. The data presented here showed that IBV T infection significantly reduced the mean mitochondrial count only in the shell gland tissue of oviduct. Although the virus seemed to have caused more effects in the shell gland, the reduced mitochondrial count could not be correlated with the expression levels of most of the genes studied except for *SDHA*. It is possible that virus multiplication in the shell gland tissue might compromise the pathways involving the synthesis of SDHA protein. Generally, viral replication alters mitochondrial permeability transition pore, oxidative balance, mitochondrial membrane potential, electron transport and energy production at the cellular level [78]. It is not clear how IBV T challenge led to the reduction of mitochondrial counts in the cells; however, in previous studies, degenerated mitochondria in IBV infected cells of shell gland tissue of laying hens have been recorded [64]. In the current study, it seems that the viral load in the shell gland tissue was very low at days 9–10 p.i. and therefore was not detectable in the shell gland tissue containing all tissue layers. However, the mean viral load was 3.55 × 10^6^ copies per gram in the epithelial mucosa (epithelial scrapings) of the shell gland region of the oviduct, indicating that shell gland had succumbed to viral infection particularly in the epithelial layer.

Based on the lack of significant differences in mitochondrial count in the cells between the challenged and control groups for the magnum and isthmus, we focused further on shell gland tissue and studied the expression level of genes involved in mitochondrial density, biogenesis and fission. The lack of any significant difference in the relative expression levels of all of the genes except *SDHA,* between the control and IBV T challenged groups, may indicate that mitochondrial function may have been enhanced and thus overall egg quality may not have been affected by fewer mitochondria in the shell gland cells of IBV T infected hens. *SDHA* functions in mitochondrial complex II, a part of the citric acid cycle and electron transport chain. A significantly lower expression level of the *SDHA* in the infected group correlates with the lower number of mitochondria. However, to confirm this correlation, further research is needed as the remaining genes studied were not affected by the virus challenge. It also seems that the virus effect on the expression level of *SDHA* was not consistent, as the mRNA was significantly lower in the virus challenge group at the 5 h but not the 15 h time-point. CS enzyme activity has been widely used as a marker for intact mitochondrial density. The activity of CS was 42% higher in the leg muscle of dominant versus subordinate male red jungle fowl, with no differences in overall muscle mass [79]. In the current study, there was a significantly higher level of *CS* mRNA at the 15 h time-point implying that mitochondrial density was higher at this time-point; however, this was not the case as the mitochondrial counts were not significantly different between the 5 and 15 h time-points of egg-shell formation. The protein encoded by the *CS* gene is a Krebs cycle enzyme that catalyses the synthesis of citrate from acetyl coenzyme A and oxaloacetate. The temporal relationships between mitochondrial biogenesis and the expression level of the genes are not clear and thus it is difficult to relate directly the expression level of such genes with mitochondrial counts at different time-points of egg-shell formation.

PGC-1α protein is a nuclear encoded protein that is localised both in the nucleus and cytoplasm [80]. In mammals, the expression of *PGC-1α* is prominent in tissues with high demands for energy [81, 82] and the mRNA level is induced in conditions such as physical exercise, fasting and exposure to cold [82]. Increase in PGC-1α protein is sufficient to induce cellular pathways important for mitochondrial biogenesis and energy metabolism [43, 44]. Mitochondrial content and oxidative capacity are different in different cells and are regulated by energy demand of a cell [83]. In a study of adenovirus infected SAOS-2 cells, the mitochondrial proteins induced by *PGC-1α* regulation resulted in increased mitochondrial content in the cells [83] measured 48 h after infection. In the current study, the expression level of *PGC-1α* was not affected by viral infection and time-points of egg-shell formation. The expression level of *PGC-1α* cannot be correlated with mitochondrial counts as the virus challenge reduced the mitochondria at both 5 and 15 h time-points of egg-shell formation. It seems that a lower number of mitochondria may not necessarily induce the up-regulation of *PGC-1α* in cells undergoing virus multiplication. Drp1 is a nuclear encoded protein that plays an important role in the fission of the outer mitochondrial membrane. In healthy cells, fission and fusion events occur to regulate mitochondrial morphology [84]. Once again, the higher expression level of *Drp1* at 15 h compared with the 5 h time-point cannot be clearly linked with higher activities of mitochondrial fission and/or fusion as most of the genes studied did not show a clear pattern of change with lower mean mitochondrial counts in IBV T challenged groups. Also, the mitochondrial counts were significantly lower at both 5 and 15 h time-points in the challenge groups, whereas no expression change of *Drp1* was observed upon virus challenge during egg-shell formation. This suggests that having a lower number of mitochondria in a cell may not necessarily relate to the expression level of *Drp1*. We consider that a spatio-temporal investigation is needed to understand the response of nuclear DNA encoded genes to mitochondrial biogenesis and fission in cells undergoing virus multiplication possibly through immunohistochemistry assays.

The objective of *Na*^*+*^*-K*^*+*^ *ATPase* quantification was to understand cell membrane potential level in the control and IBV T challenged groups and at two different time-points of egg-shell formation. *Na*^*+*^*-K*^*+*^ *ATPase* is involved in maintaining membrane excitation potential, tight junction polarity and vectorial transport in cells (reviewed in Rajasekaran et al., [85]). A higher expression level of *Na*^*+*^*-K*^*+*^ *ATPase* at 15 h compared with the 5 h time-point is an indication of higher cellular activity of the shell gland tissue during the formation of the egg-shell. A lack of significant difference in the expression level of *Na*^*+*^*-K*^*+*^ *ATPase* between the control and IBV T challenged groups indicates that viral multiplication in the cells did not alter the normal function of *Na*^*+*^*-K*^*+*^ *ATPase* and that the cells in both the groups were functioning similarly. However, an interaction of time-point and virus challenge indicated that the expression level of *Na*^*+*^*-K*^*+*^ *ATPase* was significantly affected by viral multiplication when the egg-shell formation was at its peak in the shell gland. A significant interaction between the time-point and virus challenge for *SDHA* and *Na*^*+*^*-K*^*+*^ *ATPase* indicates that the virus challenge downregulates these genes only at certain stages of egg-shell formation when the expression of the genes is high, at 5 h for *SDHA* but at 15 h for *Na*^*+*^*-K*^*+*^ *ATPase*.

## Conclusions

Taken together, the results of the current study show that the IBV T strain challenge in laying hens significantly lowered the mean mitochondrial counts only in the shell gland part of oviduct. Furthermore, the expression levels of the nuclear DNA encoded genes that are involved in mitochondrial biogenesis and/or function could not be clearly correlated with lower mean mitochondrial count and mitochondrial biogenesis. A significant difference in the expression levels of most of the genes at two different time-points of egg-shell formation showed that the mitochondrial function was more affected by egg-shell formation stages than by the viral multiplication in the cells. Further investigation is required to determine the actual turnover of mitochondria in metabolically active organs, such as laying hen oviduct, in normal and pathological conditions, and spatio-temporal relationship between mitochondrial count and expression of genes coding mitochondrial biogenesis related proteins.

## Abbreviations

CS: Citrate synthase
CYC, S: Cytochrome C, somatic
Drp1: Dynamin related protein 1
gDNA: genomic DNA
IBV: Infectious bronchitis virus
mtDNA: mitochondrial DNA
Na^+^-K^+^ATPase: sodium-potassium adenosine triphosphatase
PGC-1α: Peroxisome proliferator-activated receptor gamma coactivator 1-alpha
SDHA: Succinate dehydrogenase complex, subunit A, flavoprotein variant
